# Supplementary material for: Viral Metagenomics in the Clinical Realm: Lessons Learned from a Swiss-Wide Ring Trial
Source: Genes (Basel). 2019 Aug 28;10(9):655. doi: 10.3390/genes10090655 (PMC6770386; doi:10.3390/genes10090655)
Supplement: Supplementary file 1 [file genes-10-00655-s001.zip › revised Suppl Material/Dataset S3.pdf]

Swiss NGS viral metagenomics ring trial - submission form - increment 2

SIB Clinical Bioinformatics

Thank you for taking part in the Swiss NGS-based viral metagenomics ring trial !

Please fill in this short questionnaire to complete your submission, thank you.

There are 14 questions in this survey

General

Is the bioinformatics pipeline used for increment-2 identical to the one used in increment 1?

Increment-1: ring trial starting from samples
Increment-2: ring trial starting from FASTQ datasets (you're currently performing increment-2)

*****

Please choose **only one** of the following:

- ◯ Yes
- ◯ No
- ◯ I'm not sure I remember what we did in increment 1
- ◯ I did not participate to increment 1

We will send you an excel file with your answers from the questionnaire of increment 1. Please highlight and explain in that document any changes you may have made to the bioinformatics pipeline.

Only answer this question if the following conditions are met:
Answer was 'I'm not sure I remember what we did in increment 1' *or* 'No' at question '1 [pipeline]' (Is the bioinformatics pipeline used for increment-2 identical to the one used in increment 1? Increment-1: ring trial starting from samples Increment-2: ring trial starting from FASTQ datasets (you're currently performing increment-2))

You had the possibility to identify the viruses based on the SIB-provided common database, and also using your own in-house database.

Which database(s) did you use to identify the viruses in the samples?

*****

Please choose all that apply and provide a comment:

- ☐ SIB-provided common database
- ☐ Own, in-house database

Suppose you have 1 urgent FASTQ dataset to be analyzed using metagenomics. In how many hours could you provide a report for pathogen identification (i.e. duration <u>from FASTQ-processing</u> to report, under emergency conditions, no budget limitations)?

*****

Please write your answer here:

Please write here any general comments on the ring trial, thank you.

Please write your answer here:

Reads classification

Were reads quality filtered before conducting the analysis? *

Only answer this question if the following conditions are met:
Answer was 'I did not participate to increment 1' at question '1 [pipeline]' (Is the bioinformatics pipeline used for increment-2 identical to the one used in increment 1? Increment-1: ring trial starting from samples Increment-2: ring trial starting from FASTQ datasets (you're currently performing increment-2))

Please choose **only one** of the following:

- ○ Yes
- ○ No
- ○ I don't know

Please specify how reads were quality filtered before conducting the analysis.

Only answer this question if the following conditions are met:
Answer was 'Yes' at question '6 [preproc0]' (Were reads quality filtered before conducting the analysis?) *and* Answer was 'I did not participate to increment 1' at question '1 [pipeline]' (Is the bioinformatics pipeline used for increment-2 identical to the one used in increment 1? Increment-1: ring trial starting from samples Increment-2: ring trial starting from FASTQ datasets (you're currently performing increment-2))

Please choose **all** that apply:

- ☐ cutadapt
- ☐ trimmomatic
- ☐ PrinSeq
- ☐ FastQC
- ☐ fastq-mcf
- ☐ pear
- ☐ Other: ____________

How did you perform removal of reads from host origin (DNA or RNA)? *

Only answer this question if the following conditions are met:
Answer was 'I did not participate to increment 1' at question '1 [pipeline]' (Is the bioinformatics pipeline used for increment-2 identical to the one used in increment 1? Increment-1: ring trial starting from samples Increment-2: ring trial starting from FASTQ datasets (you're currently performing increment-2))

Please choose **only one** of the following:

- ○ First remove reads of host origin. Then map remaining reads to a target library.
- ○ First map reads against a target library. Then, among the remaining reads, remove those of host origin.
- ○ Classify reads simultaneously into several categories (e.g. host vs. non-host, or viral vs. bacterial vs other).
- ○ Other ____________

What type of approach did you use for taxonomic classification? *

Only answer this question if the following conditions are met:
Answer was 'I did not participate to increment 1' at question '1 [pipeline]' (Is the bioinformatics pipeline used for increment-2 identical to the one used in increment 1? Increment-1: ring trial starting from samples Increment-2: ring trial starting from FASTQ datasets (you're currently performing increment-2))

Please choose **all** that apply:

- ☐ Mapping-based (alignment)
- ☐ k-mer based
- ☐ De novo assembly of all reads prior to mapping
- ☐ De novo assembly of unclassified reads only
- ☐ Other: ____________

Alignment tool

Only answer this question if the following conditions are met:
Answer was at question '9 [48c]' (What type of approach did you use for taxonomic classification?) *and* Answer was 'I did not participate to increment 1' at question '1 [pipeline]' (Is the bioinformatics pipeline used for increment-2 identical to the one used in increment 1? Increment-1: ring trial starting from samples Increment-2: ring trial starting from FASTQ datasets (you're currently performing increment-2))

Please choose **all** that apply:

- ☐ bwa mem
- ☐ bwa aln
- ☐ bowtie
- ☐ bowtie2
- ☐ SMALT
- ☐ STAR
- ☐ NovoAlign
- ☐ DIAMOND
- ☐ blastn
- ☐ blastp
- ☐ blastx
- ☐ SNAP
- ☐ Clustal
- ☐ Mafft
- ☐ Muscle
- ☐ Other: ____________

K-mer based tool

Only answer this question if the following conditions are met:
Answer was at question '9 [48c]' (What type of approach did you use for taxonomic classification?) *and* Answer was 'I did not participate to increment 1' at question '1 [pipeline]' (Is the bioinformatics pipeline used for increment-2 identical to the one used in increment 1? Increment-1: ring trial starting from samples Increment-2: ring trial starting from FASTQ datasets (you're currently performing increment-2))

Please choose **all** that apply:

- ☐ In-house
- ☐ Kraken
- ☐ CLARK
- ☐ Taxonomer
- ☐ Other: ____________

De novo assembly tool

Only answer this question if the following conditions are met:

-------- Scenario 1 --------

Answer was at question '9 [48c]' (What type of approach did you use for taxonomic classification?) *and* Answer was 'I did not participate to increment 1' at question '1 [pipeline]' (Is the bioinformatics pipeline used for increment-2 identical to the one used in increment 1? Increment-1: ring trial starting from samples Increment-2: ring trial starting from FASTQ datasets (you're currently performing increment-2))

-------- or Scenario 2 --------

Answer was at question '9 [48c]' (What type of approach did you use for taxonomic classification?)

Please choose **all** that apply:

- ☐ In-house
- ☐ metaVelvet
- ☐ meta-IDBA
- ☐ metaSPAdes
- ☐ Other: ____________

Data submission

I have named all the files using my lab pipeline letter and the sample number, e.g. B2.bam, for the BAM of sample 2 analyzed with lab pipeline B. In addition, for the in-silico datasets, I have specified the sequencing parameters as well (e.g. B2_1x100.bam).

*

Please choose **only one** of the following:

- Yes
- No

Submit data

A couple of weeks before the end of the trial, you will receive SWITCH FileSender vouchers to submit your data (or earlier if you wish, please send an email to aitana.lebrand@sib.swiss, as vouchers are only valid for 2 weeks).

For each sample analyzed, the submitted data would consist of the following:

1. BAM file, BGA file or TSV file (e.g. for Kraken)
2. Metrics file in TSV format with the following columns:
   Virus | database_ID | Number_of_reads | percent_coverage | genome_size | report | comment
   - The percent coverage is defined as the fraction of the genome that is covered by at least one read.
   - The genome size should also be provided to enable the computation of other metrics by SIB.
   - "report" column should be set as "1" if you would report this virus to clinicians, and "0" otherwise. You may add a comment regarding reported status.
3. For one of the samples, please submit a clinical and a lab report, so we can compare and discuss reporting practices.

I have read carefully the above explanations.

*

Only answer this question if the following conditions are met:
Answer was 'Yes' *or* 'No' at question '13 [wa]' (I have named all the files using my lab pipeline letter and the sample number, e.g. B2.bam, for the BAM of sample 2 analyzed with lab pipeline B. In addition, for the in-silico datasets, I have specified the sequencing parameters as well (e.g. B2_1x100.bam).)

Please choose **only one** of the following:

- Yes
- No

Thank you very much for your participation, your answers and data have been recorded.

22-10-2018 – 11:56

Submit your survey.
Thank you for completing this survey.
