# Supplementary material for: Viral Metagenomics in the Clinical Realm: Lessons Learned from a Swiss-Wide Ring Trial
Source: Genes (Basel). 2019 Aug 28;10(9):655. doi: 10.3390/genes10090655 (PMC6770386; doi:10.3390/genes10090655)
Supplement: Supplementary file 1 [file genes-10-00655-s001.zip › revised Suppl Material/Figures S3-7.pdf]

Figure S3

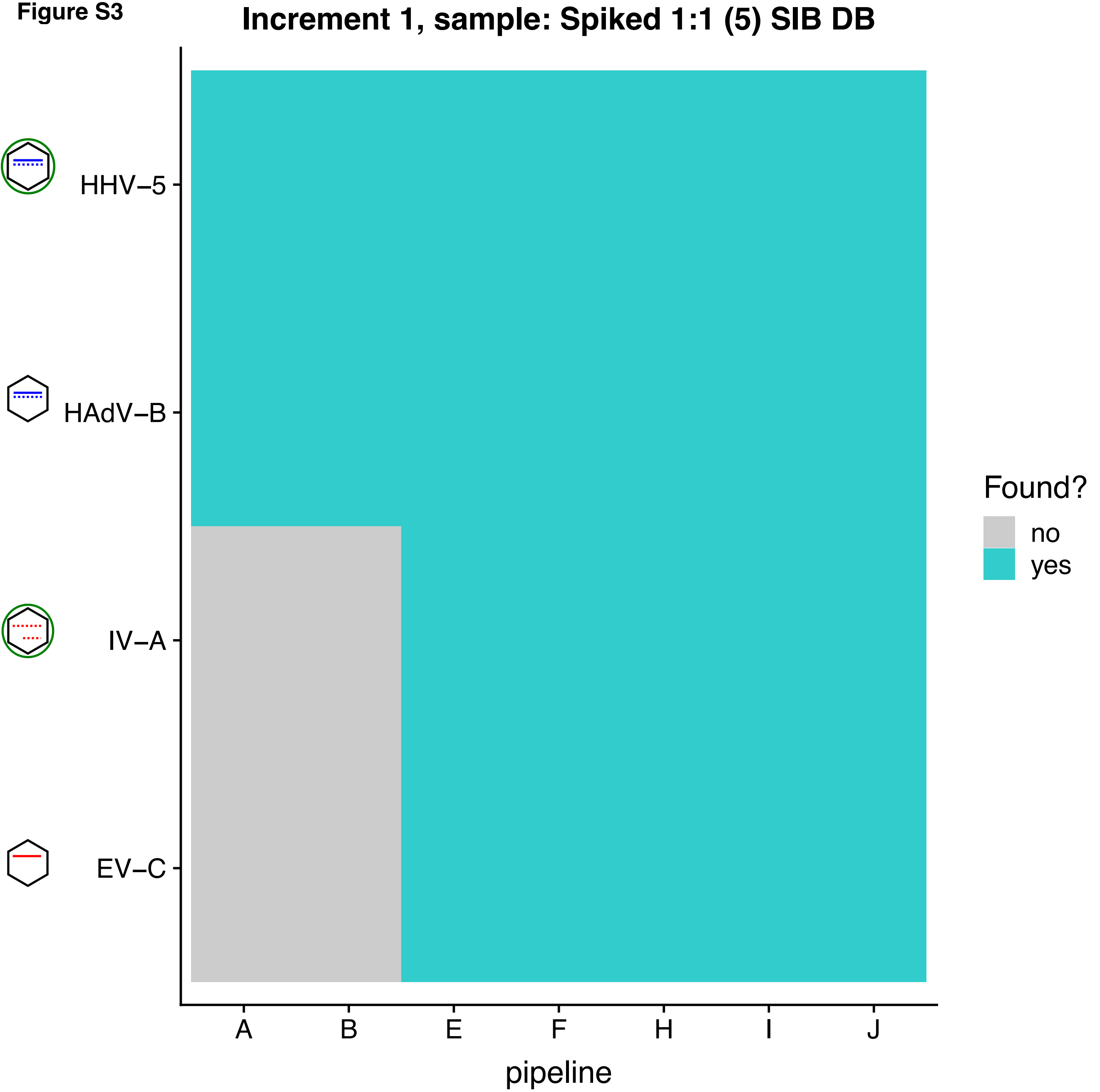
Increment 1, sample: Spiked 1:1 (5) SIB DB
HHV−5
HAdV−B
IV−A
EV−C
A
B
E
F
H
I
J
pipeline
Found?
no
yes

Figure S4

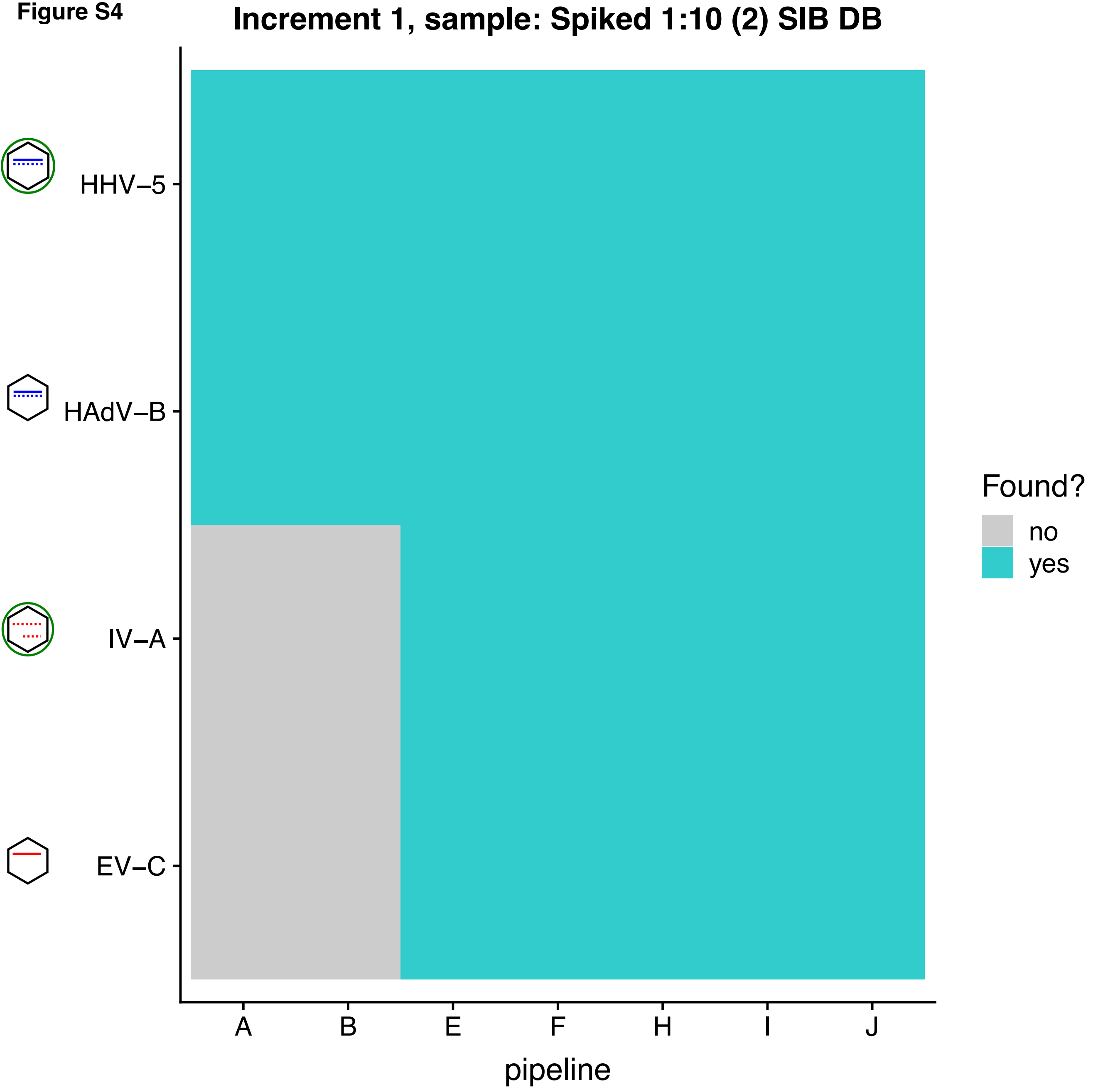
Increment 1, sample: Spiked 1:10 (2) SIB DB
HHV−5
HAdV−B
IV−A
EV−C
A
B
E
F
H
I
J
pipeline
Found?
no
yes

Figure S5

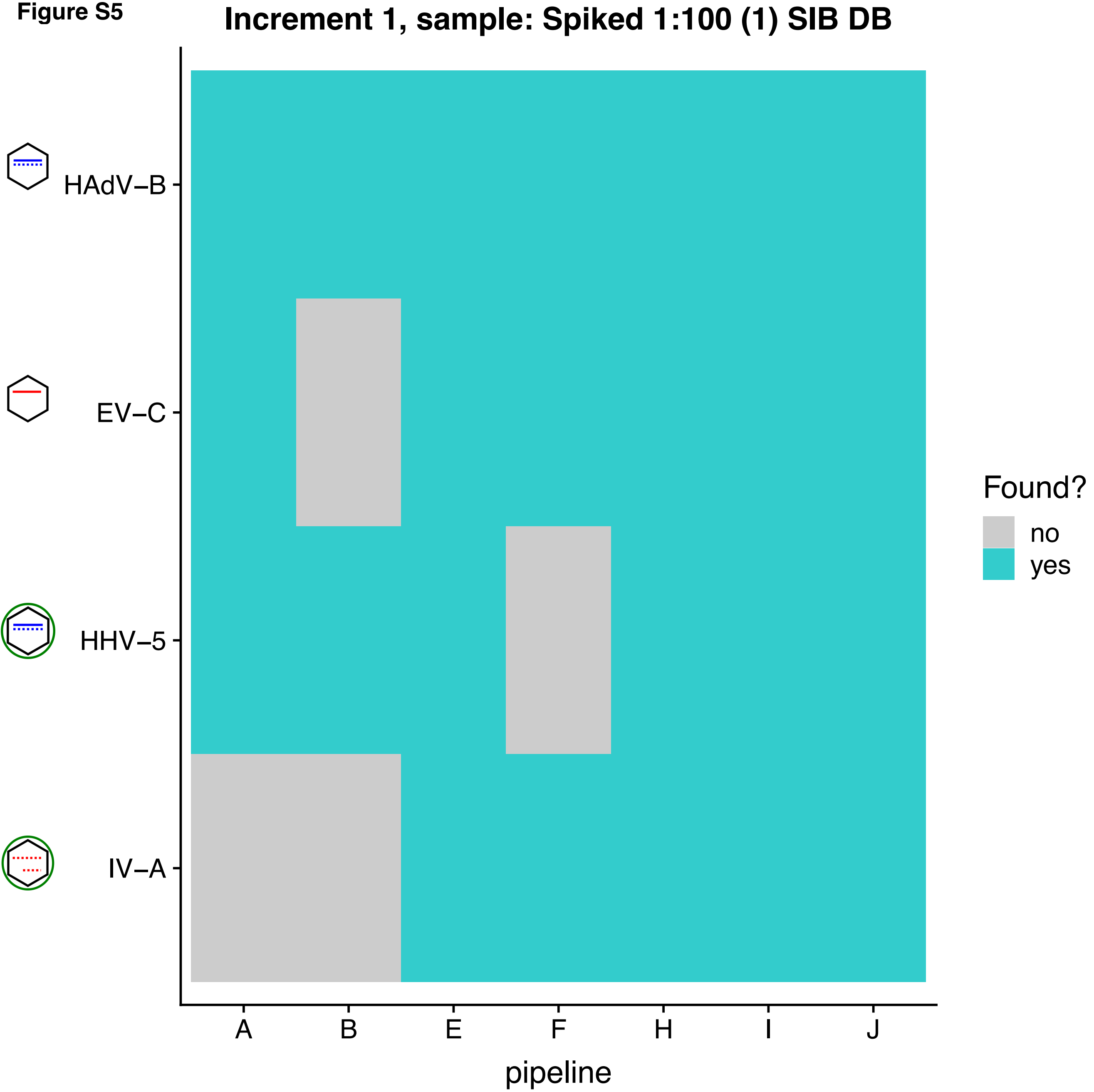
Increment 1, sample: Spiked 1:100 (1) SIB DB
HAdV–B
EV–C
HHV–5
IV–A
A
B
E
F
H
I
J
pipeline
Found?
no
yes

Figure S6

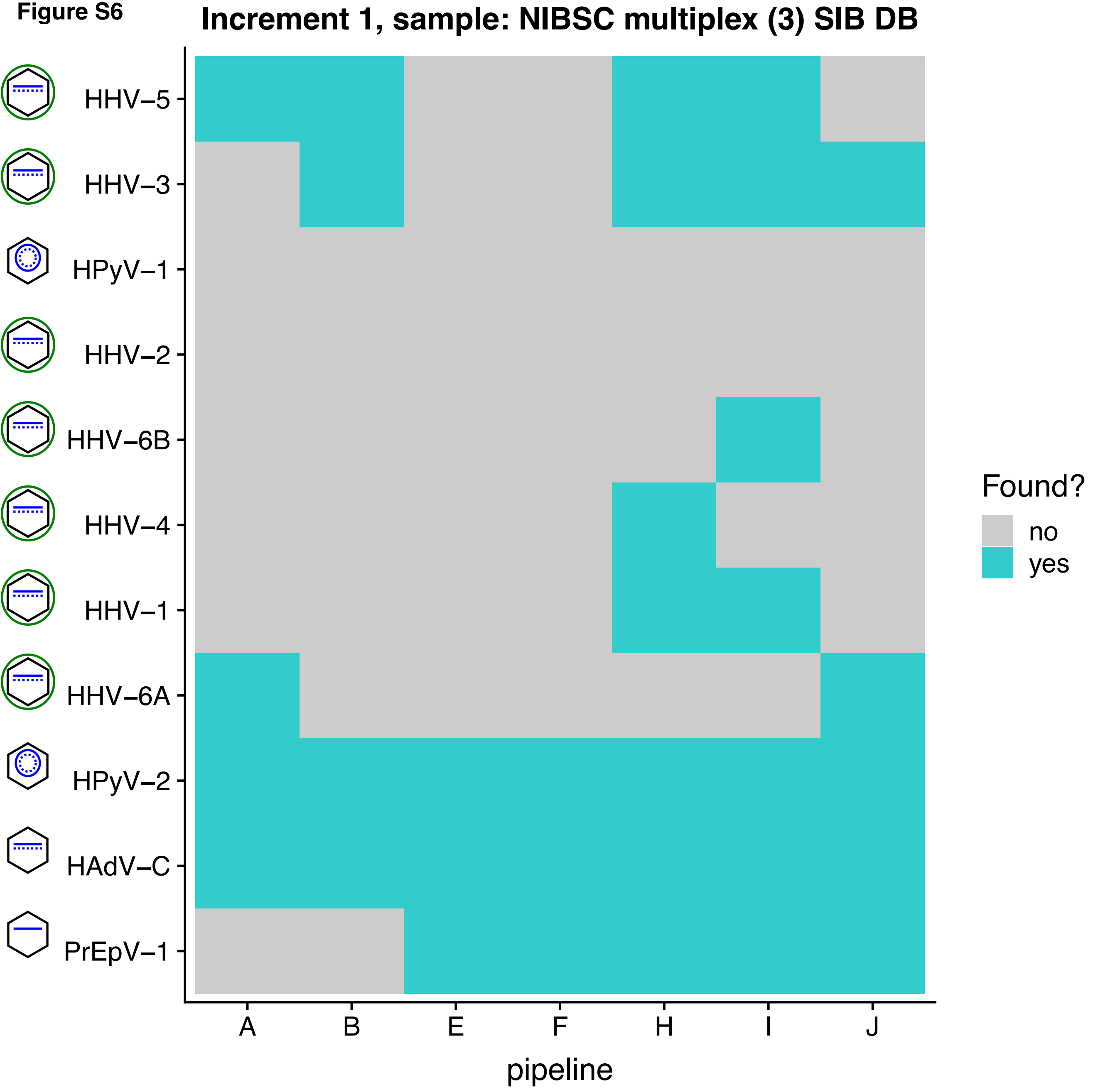
Increment 1, sample: NIBSC multiplex (3) SIB DB
HHV−5
HHV−3
HPyV−1
HHV−2
HHV−6B
HHV−4
HHV−1
HHV−6A
HPyV−2
HAdV−C
PrEpV−1
A
B
E
F
H
I
J
pipeline
Found?
no
yes

Figure S7

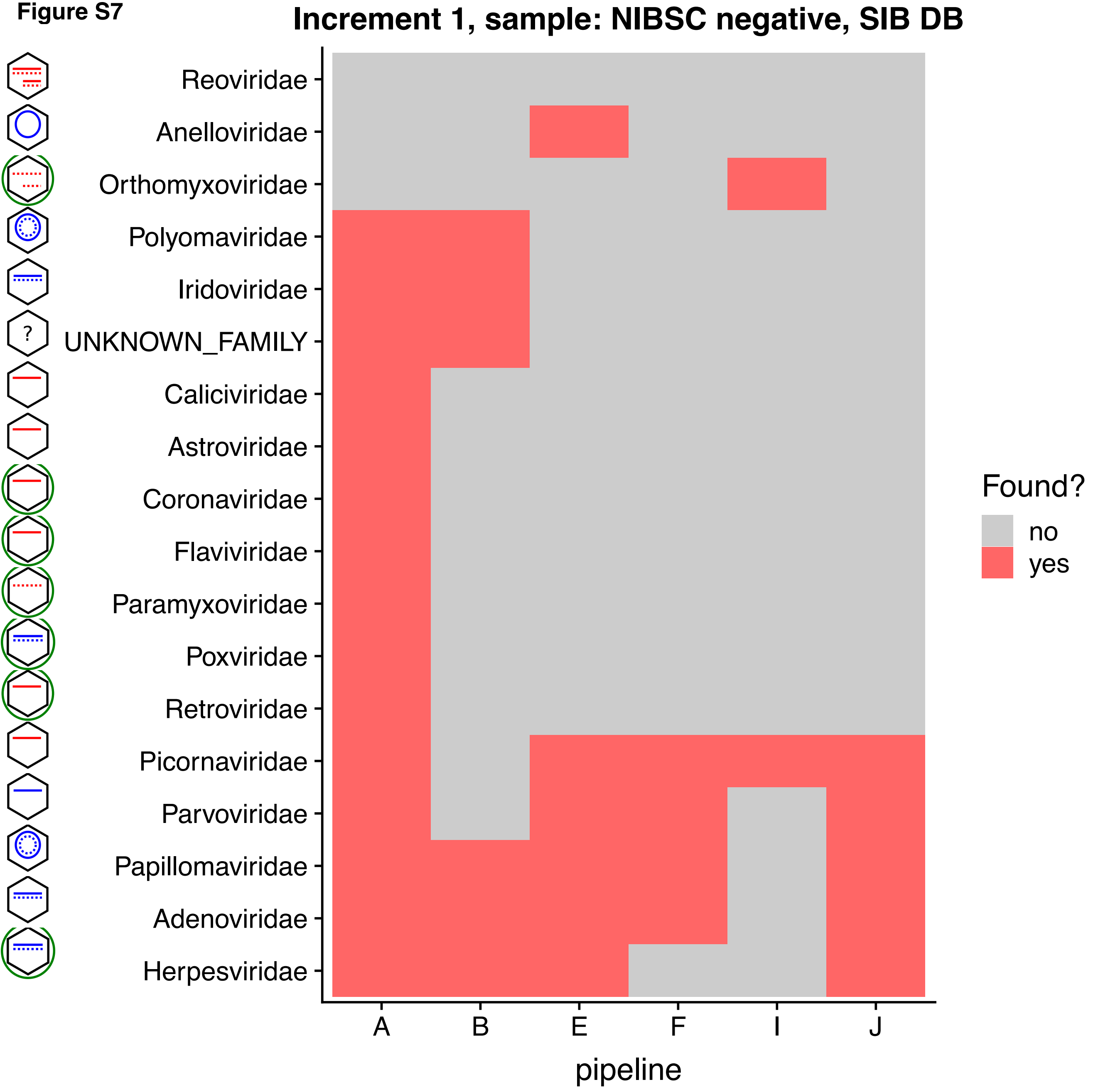
Increment 1, sample: NIBSC negative, SIB DB
Reoviridae
Anelloviridae
Orthomyxoviridae
Polyomaviridae
Iridoviridae
UNKNOWN_FAMILY
Caliciviridae
Astroviridae
Coronaviridae
Flaviviridae
Paramyxoviridae
Poxviridae
Retroviridae
Picornaviridae
Parvoviridae
Papillomaviridae
Adenoviridae
Herpesviridae
?
A
B
E
F
I
J
pipeline
Found?
no
yes
