# Supplementary material for: Viral Metagenomics in the Clinical Realm: Lessons Learned from a Swiss-Wide Ring Trial
Source: Genes (Basel). 2019 Aug 28;10(9):655. doi: 10.3390/genes10090655 (PMC6770386; doi:10.3390/genes10090655)
Supplement: Supplementary file 1 [file genes-10-00655-s001.zip › revised Suppl Material/Figures S8-19.pdf]

Figure S8

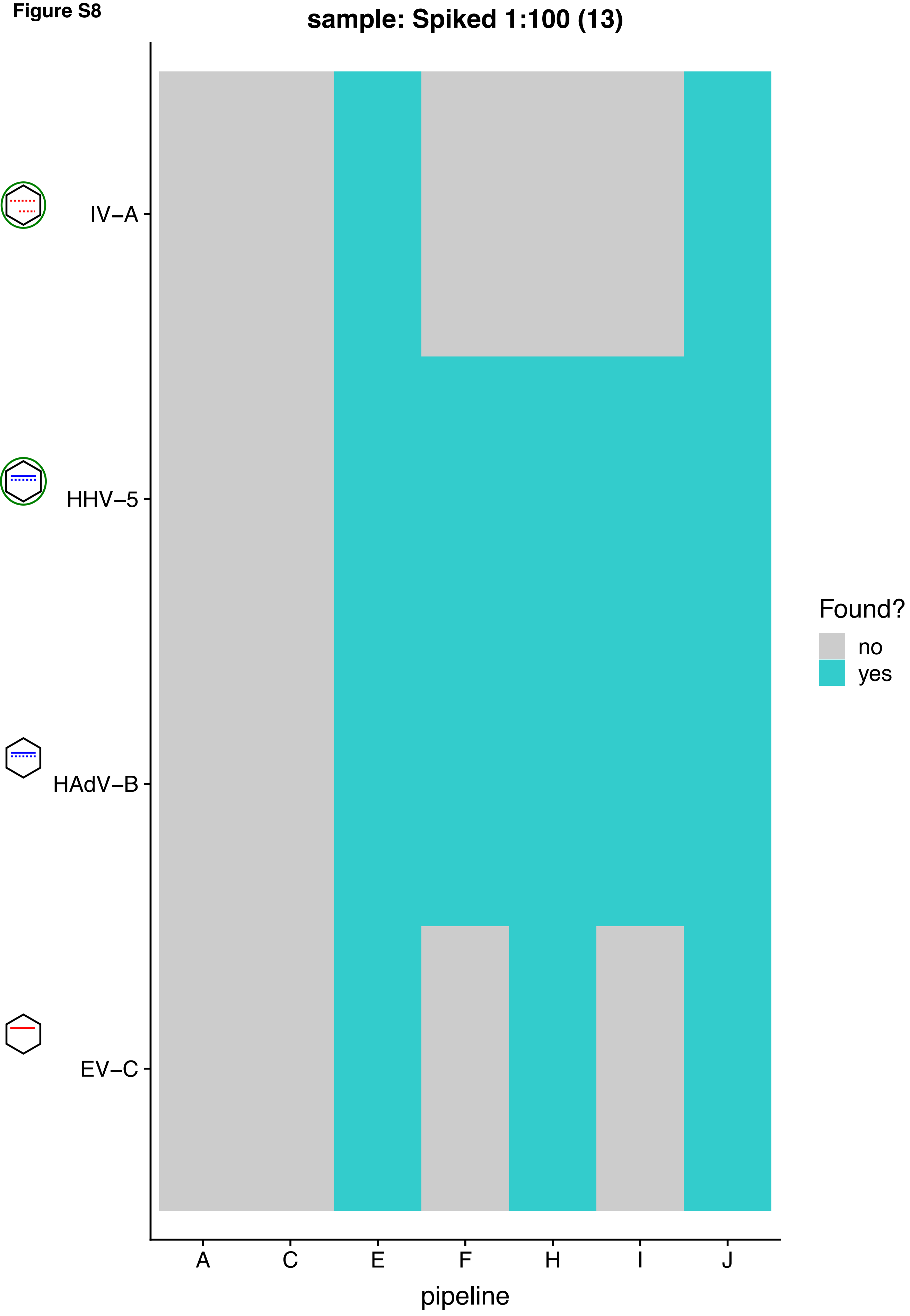
sample: Spiked 1:100 (13)
IV–A
HHV–5
HAdV–B
EV–C
A
C
E
F
H
I
J
pipeline
Found?
no
yes

Figure S9

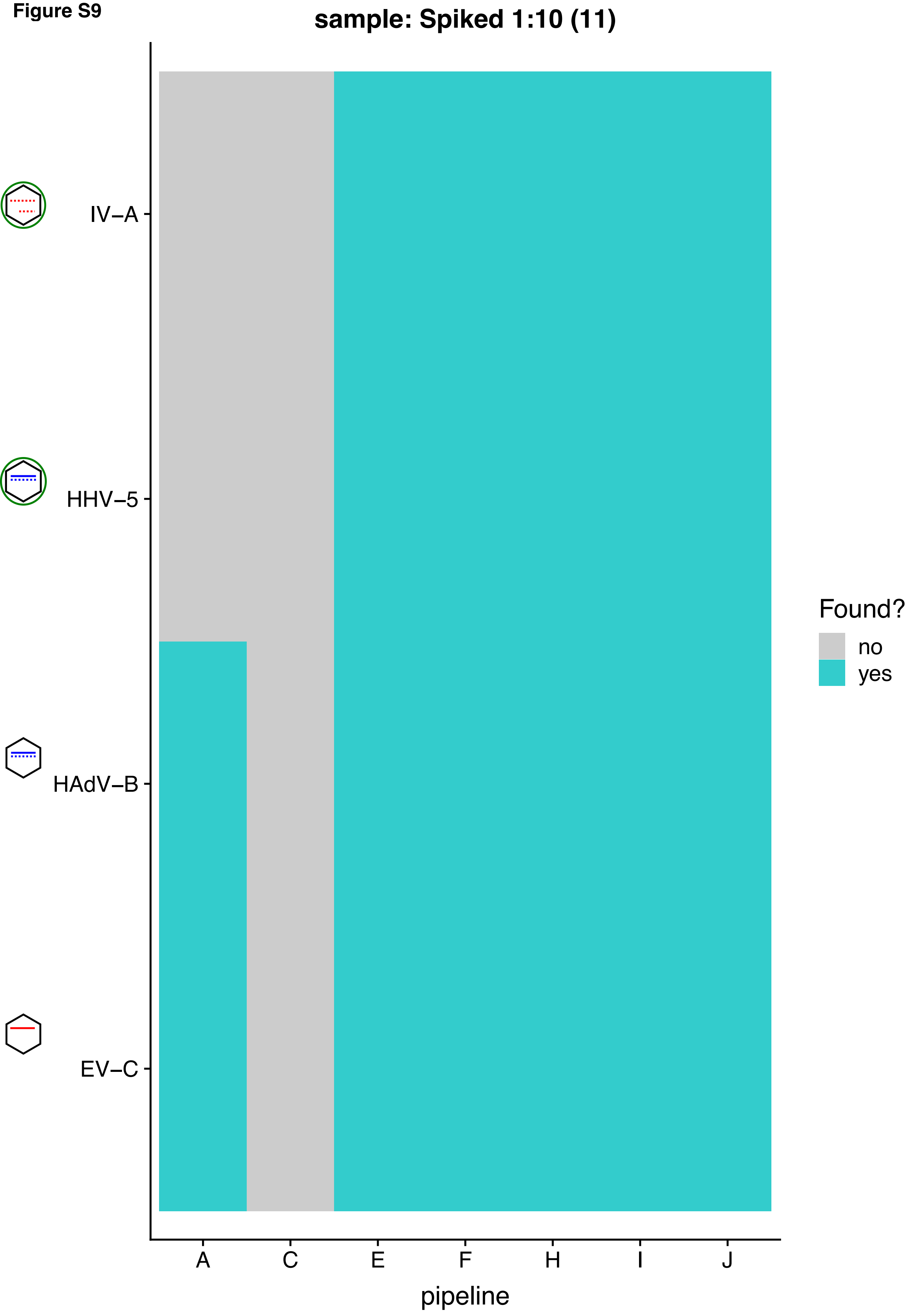
sample: Spiked 1:10 (11)
IV–A
HHV–5
HAdV–B
EV–C
A
C
E
F
H
I
J
pipeline
Found?
no
yes

Figure S10

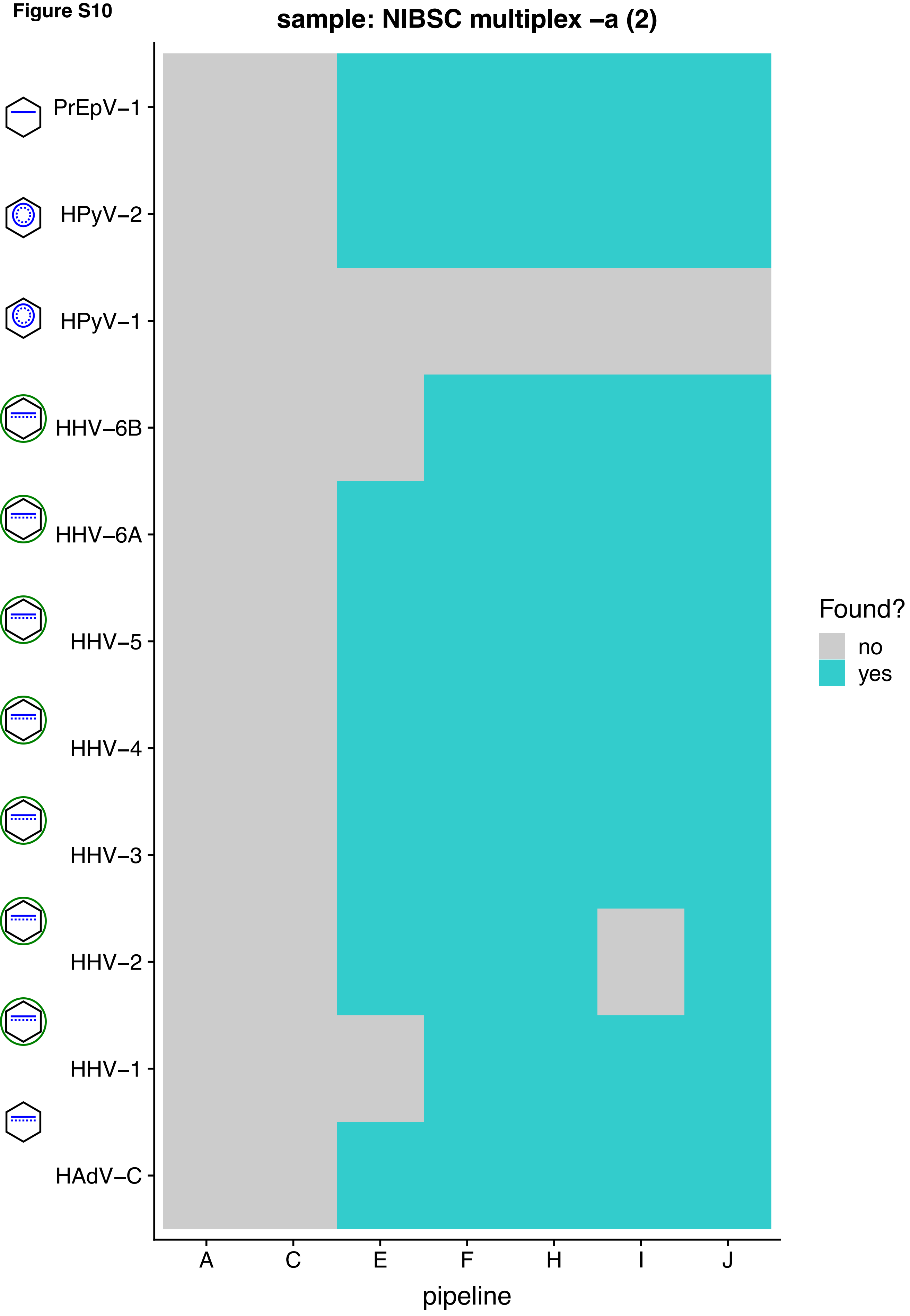
sample: NIBSC multiplex –a (2)
PrEpV–1
HPyV–2
HPyV–1
HHV–6B
HHV–6A
HHV–5
HHV–4
HHV–3
HHV–2
HHV–1
HAdV–C
A
C
E
F
H
I
J
pipeline
Found?
no
yes

Figure S11

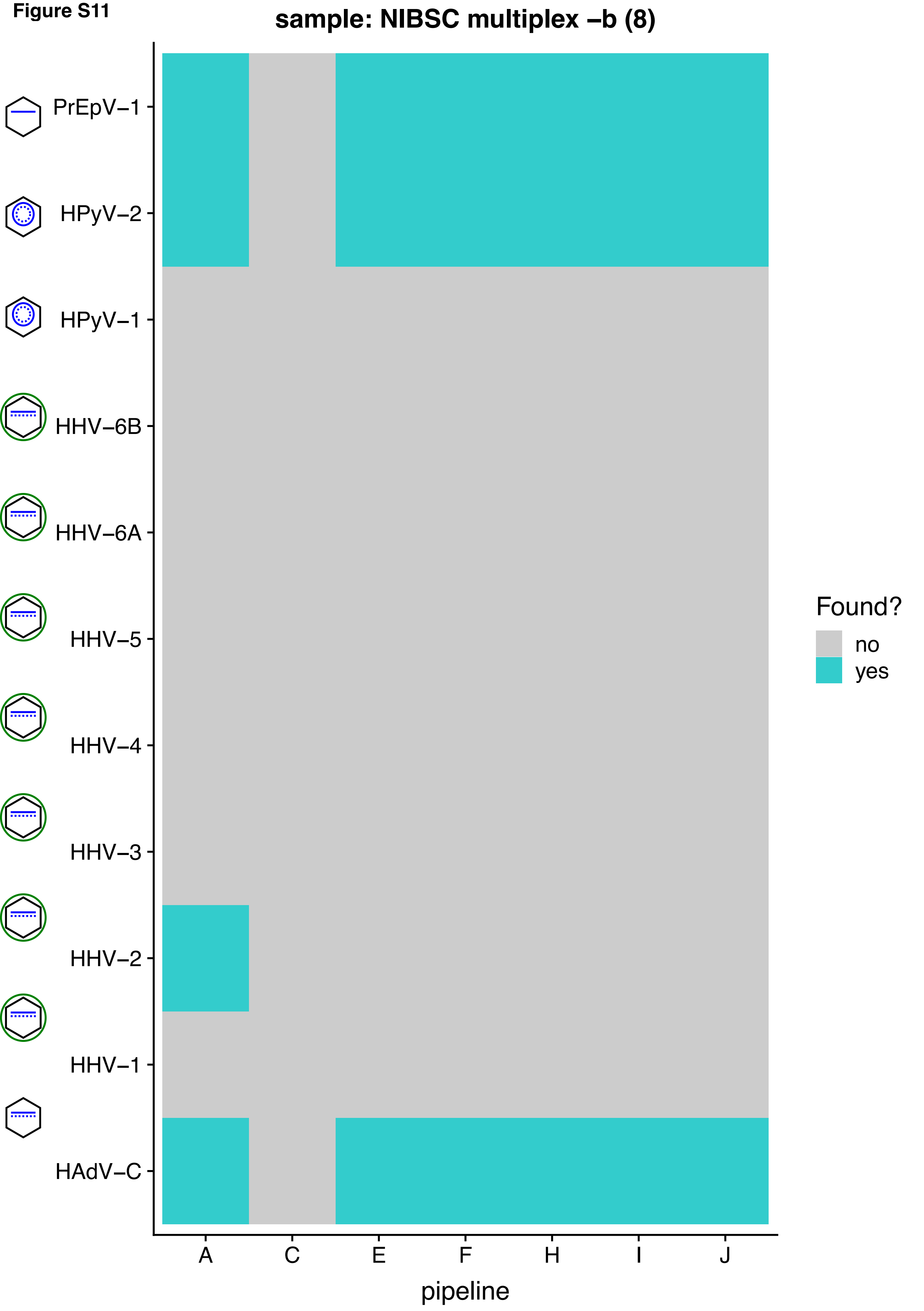
sample: NIBSC multiplex −b (8)
PrEpV−1
HPyV−2
HPyV−1
HHV−6B
HHV−6A
HHV−5
HHV−4
HHV−3
HHV−2
HHV−1
HAdV−C
A
C
E
F
H
I
J
pipeline
Found?
no
yes

Figure S12

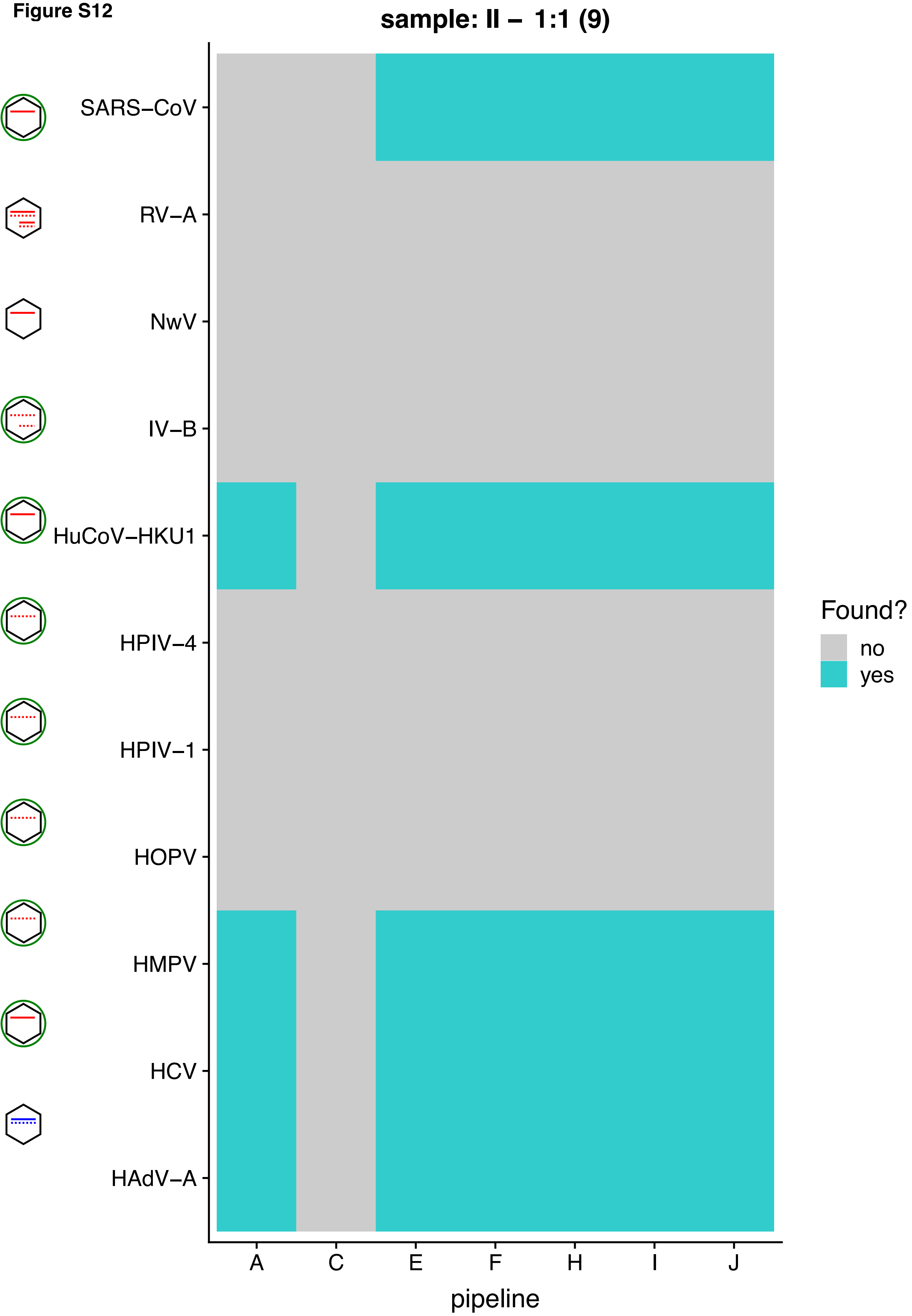
sample: II – 1:1 (9)
SARS–CoV
RV–A
NwV
IV–B
HuCoV–HKU1
HPIV–4
HPIV–1
HOPV
HMPV
HCV
HAdV–A
A
C
E
F
H
I
J
pipeline
Found?
no
yes

Figure S13

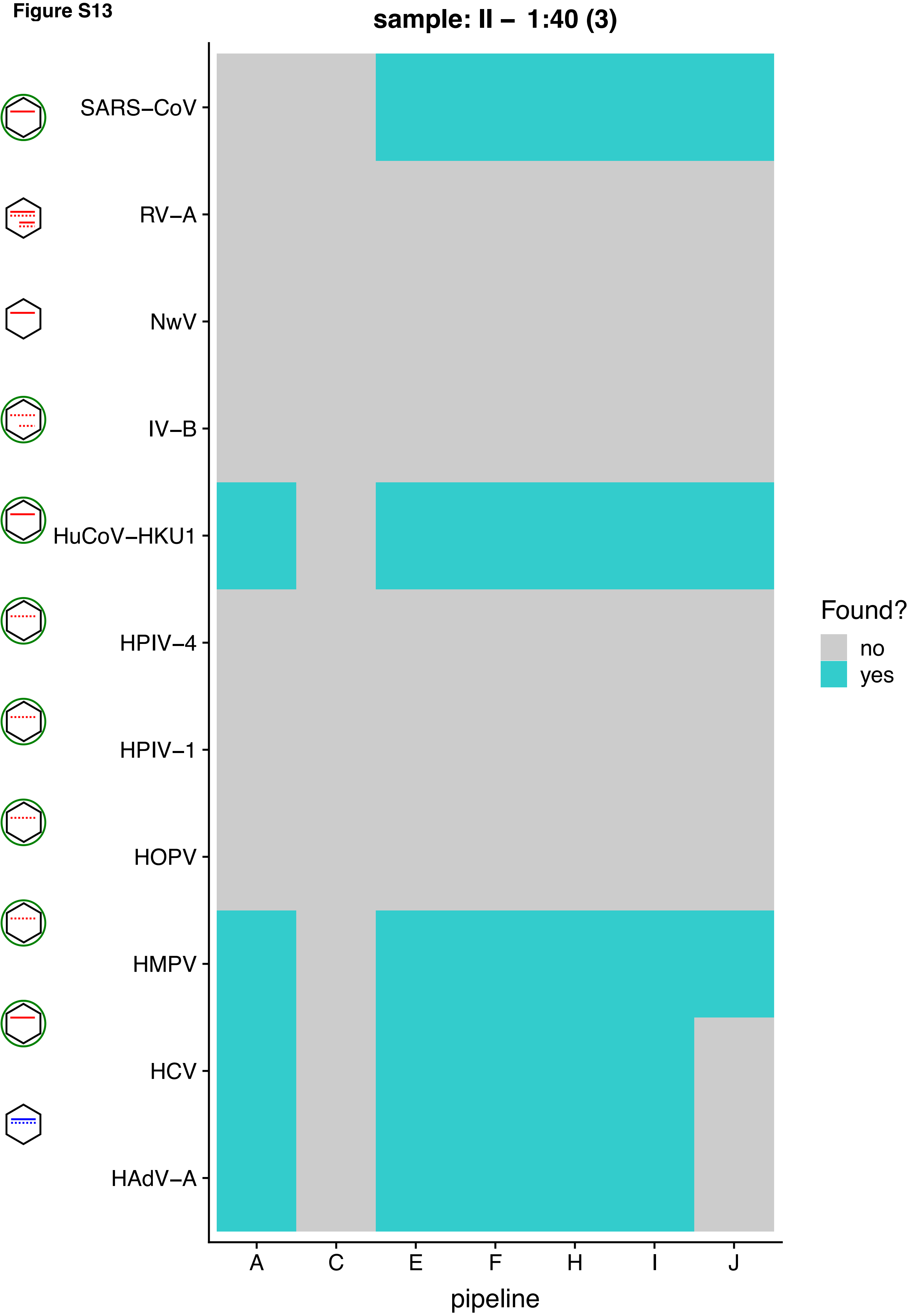

sample: II – 1:40 (3)
SARS–CoV
RV–A
NwV
IV–B
HuCoV–HKU1
HPIV–4
HPIV–1
HOPV
HMPV
HCV
HAdV–A
A
C
E
F
H
I
J
pipeline
Found?
no
yes

Figure S14

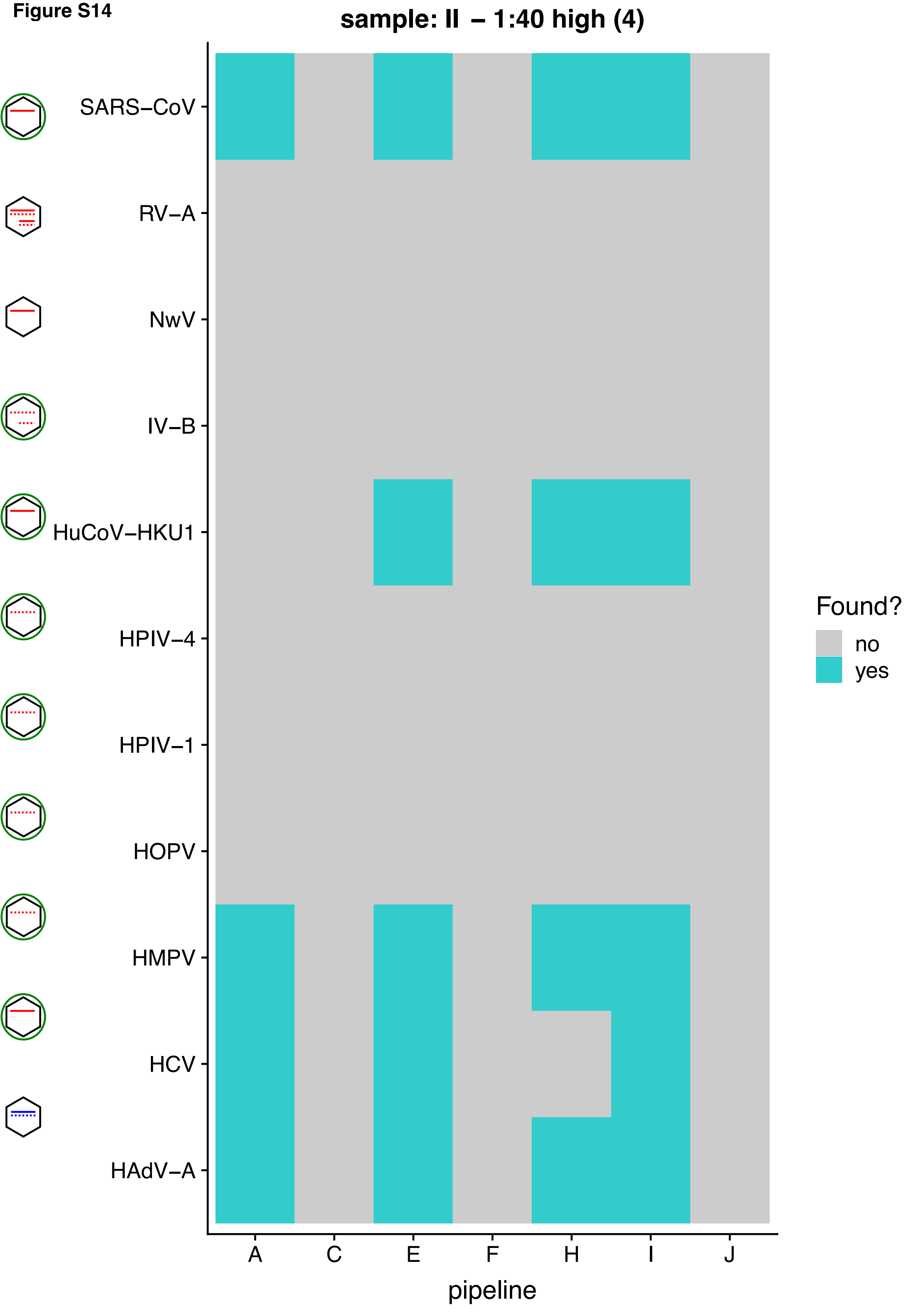
sample: II – 1:40 high (4)
SARS–CoV
RV–A
NwV
IV–B
HuCoV–HKU1
HPIV–4
HPIV–1
HOPV
HMPV
HCV
HAdV–A
A
C
E
F
H
I
J
pipeline
Found?
no
yes

Figure S15

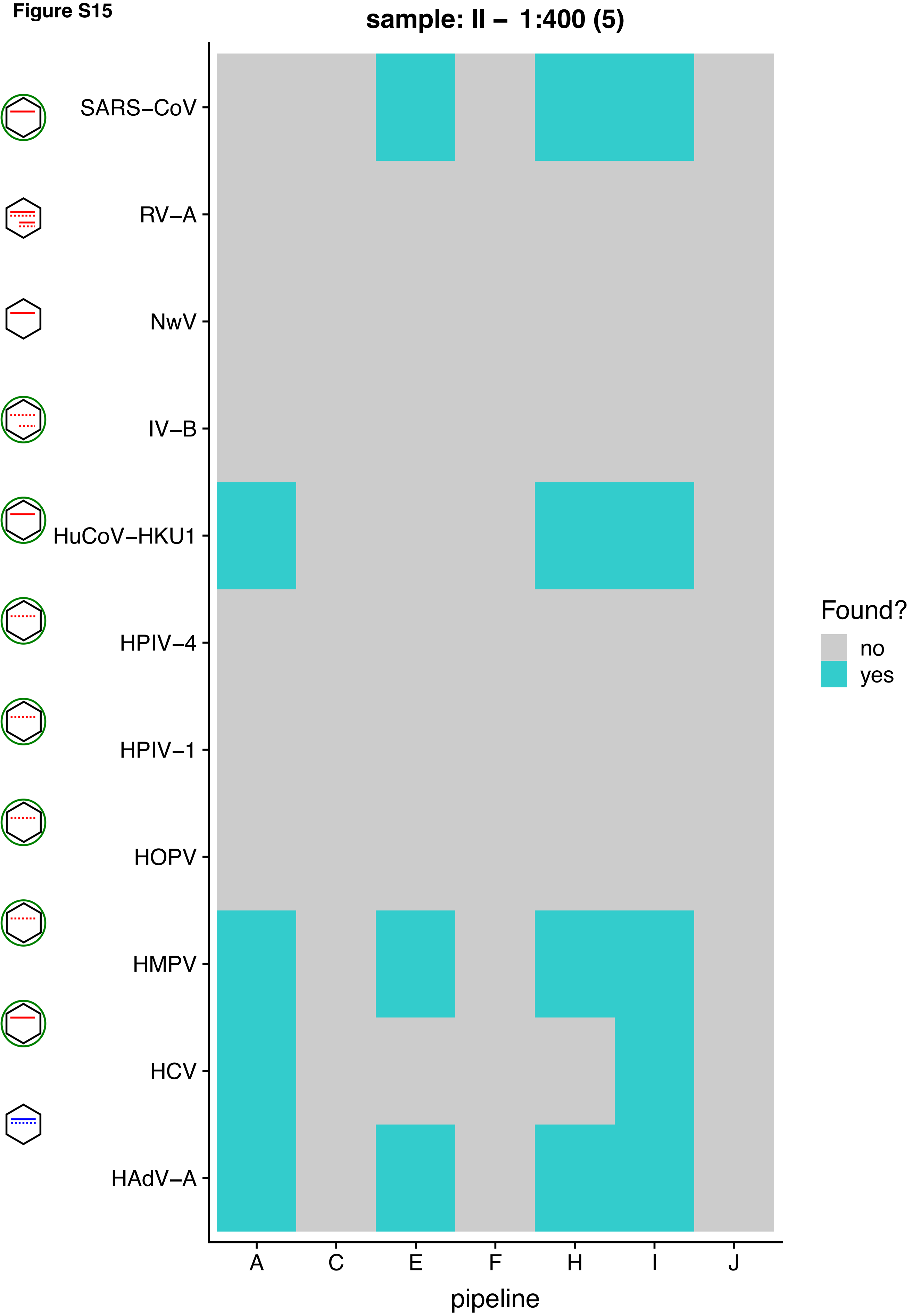

sample: II – 1:400 (5)
SARS–CoV
RV–A
NwV
IV–B
HuCoV–HKU1
HPIV–4
HPIV–1
HOPV
HMPV
HCV
HAdV–A
A
C
E
F
H
I
J
pipeline
Found?
no
yes

Figure S16

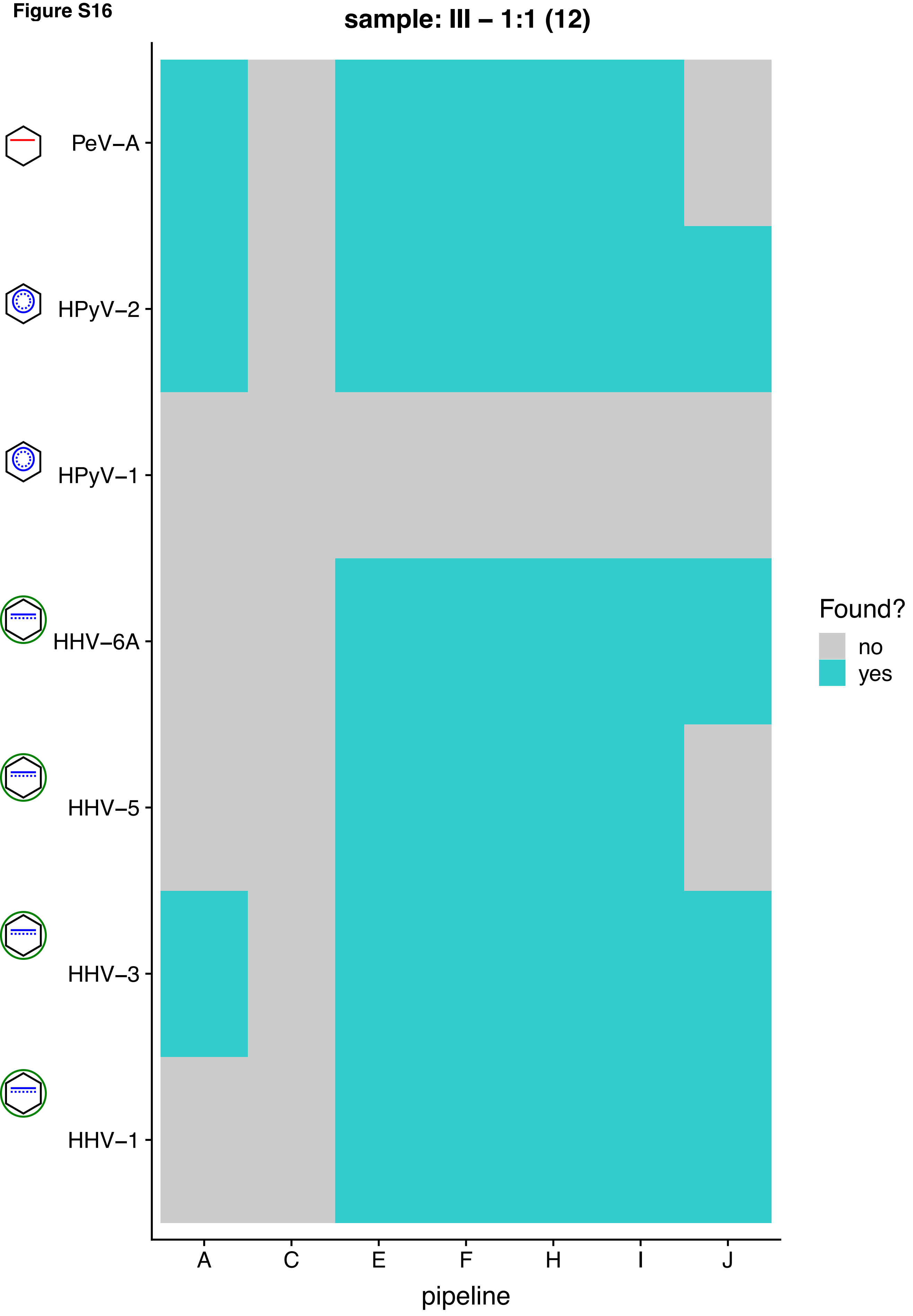
sample: III – 1:1 (12)
PeV–A
HPyV–2
HPyV–1
HHV–6A
HHV–5
HHV–3
HHV–1
A
C
E
F
H
I
J
pipeline
Found?
no
yes

Figure S17

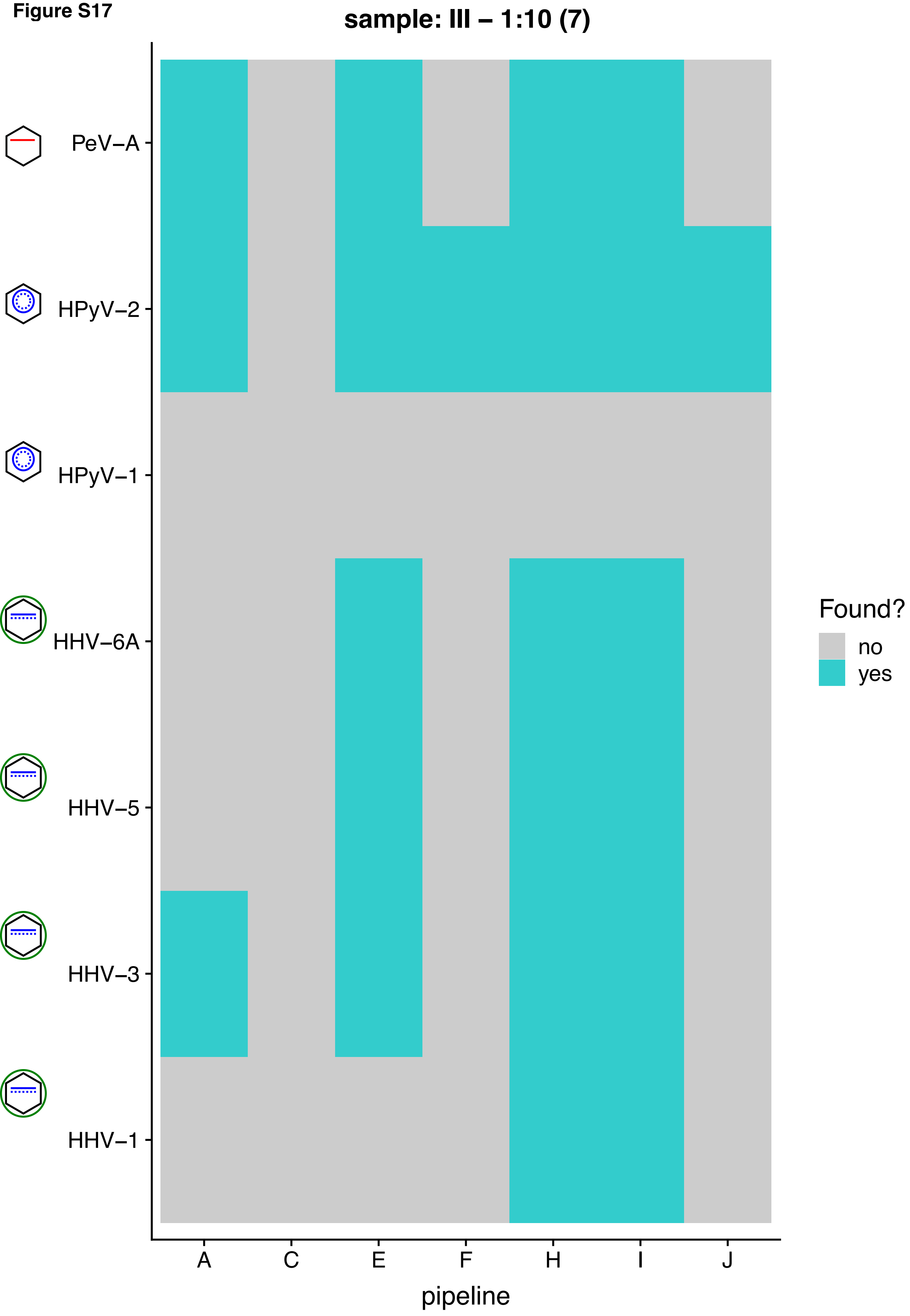
sample: III – 1:10 (7)
PeV–A
HPyV–2
HPyV–1
HHV–6A
HHV–5
HHV–3
HHV–1
A
C
E
F
H
I
J
pipeline
Found?
no
yes

Figure S18

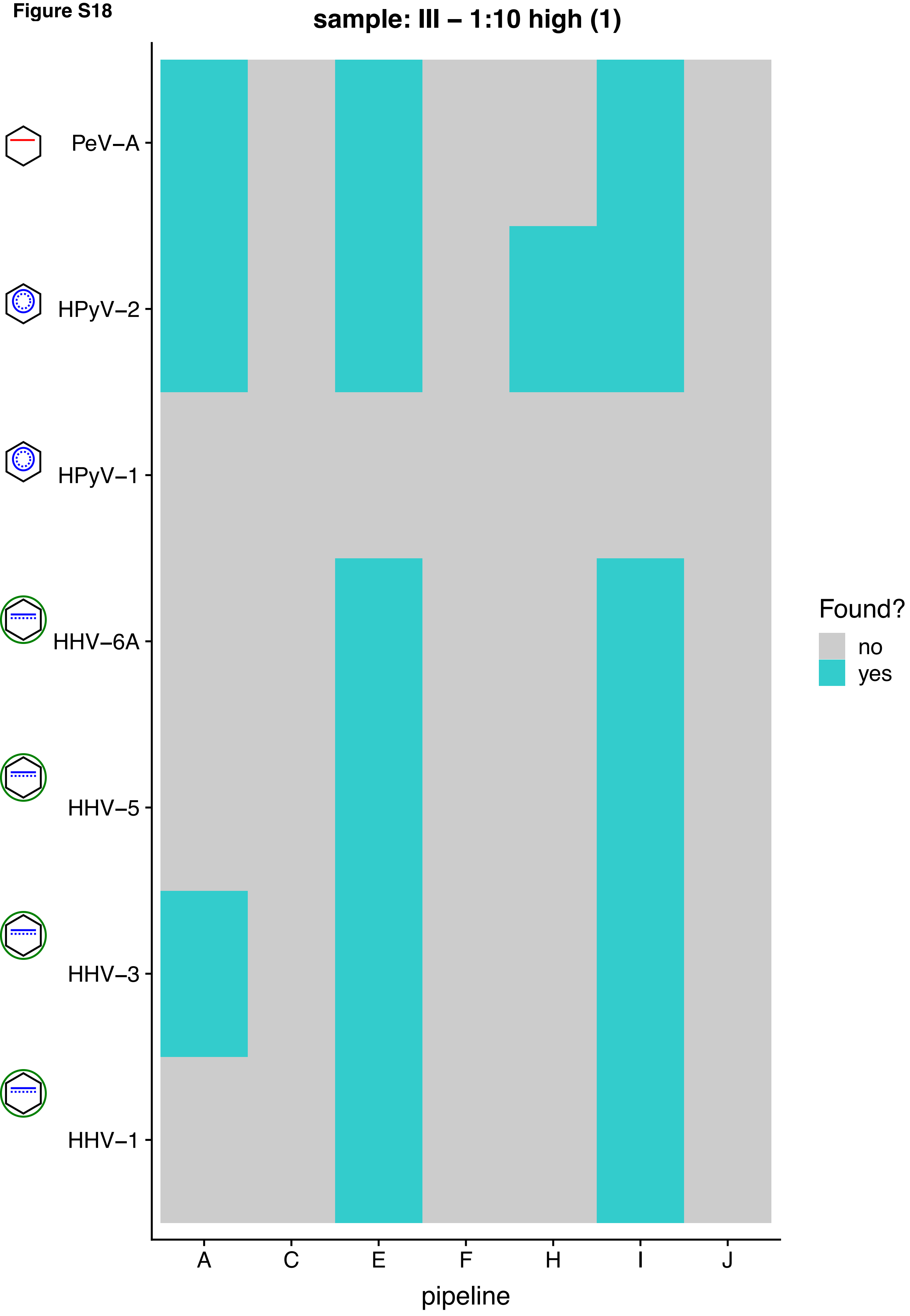
sample: III – 1:10 high (1)
PeV–A
HPyV–2
HPyV–1
HHV–6A
HHV–5
HHV–3
HHV–1
A
C
E
F
H
I
J
pipeline
Found?
no
yes

Figure S19

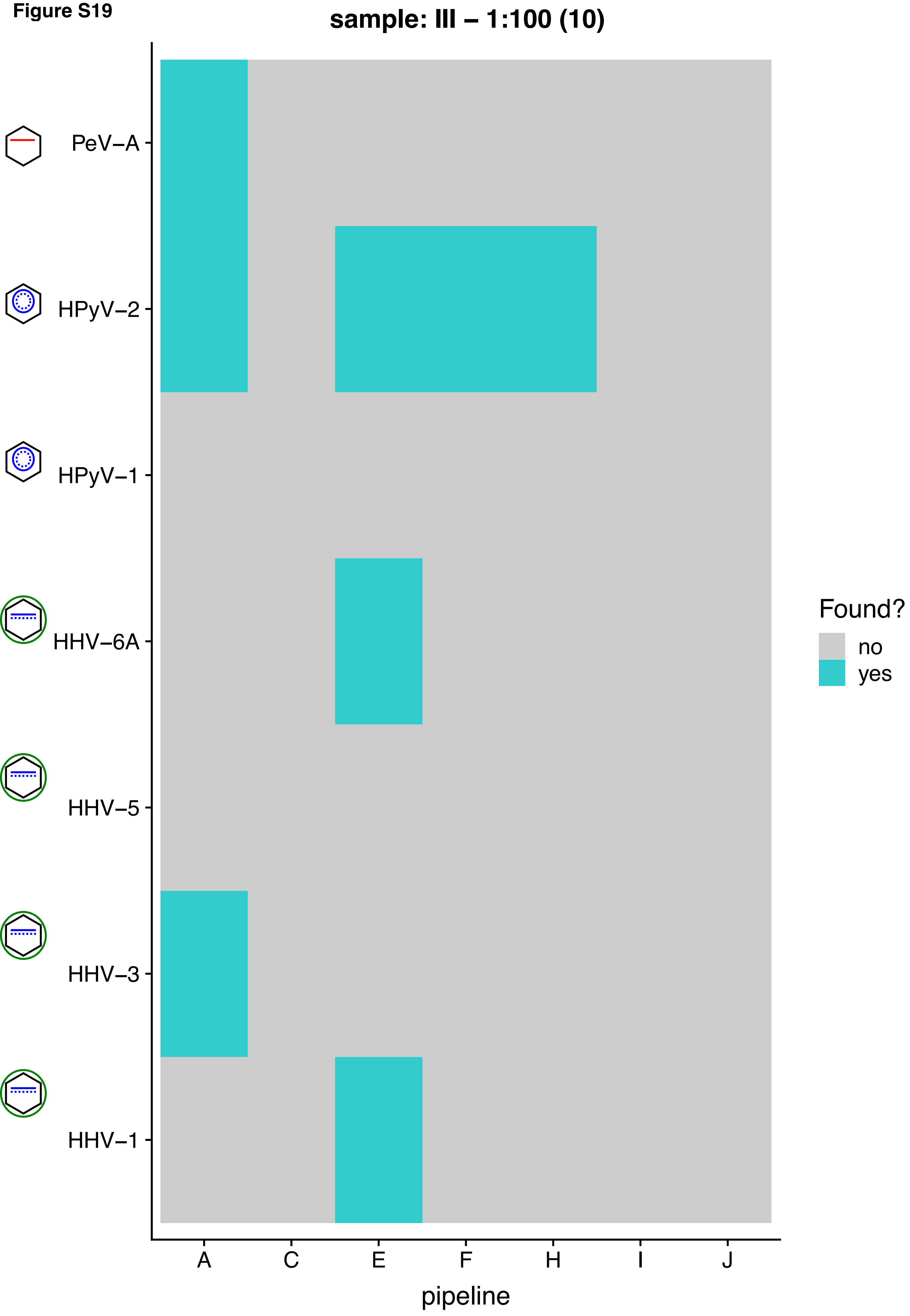

sample: III – 1:100 (10)
PeV–A
HPyV–2
HPyV–1
HHV–6A
HHV–5
HHV–3
HHV–1
A
C
E
F
H
I
J
pipeline
Found?
no
yes
