# Supplementary figures and images for: Viral Metagenomics in the Clinical Realm: Lessons Learned from a Swiss-Wide Ring Trial
Source: Genes (Basel). 2019 Aug 28;10(9):655. doi: 10.3390/genes10090655 (PMC6770386; doi:10.3390/genes10090655)

# Sensitivity by database and pipeline

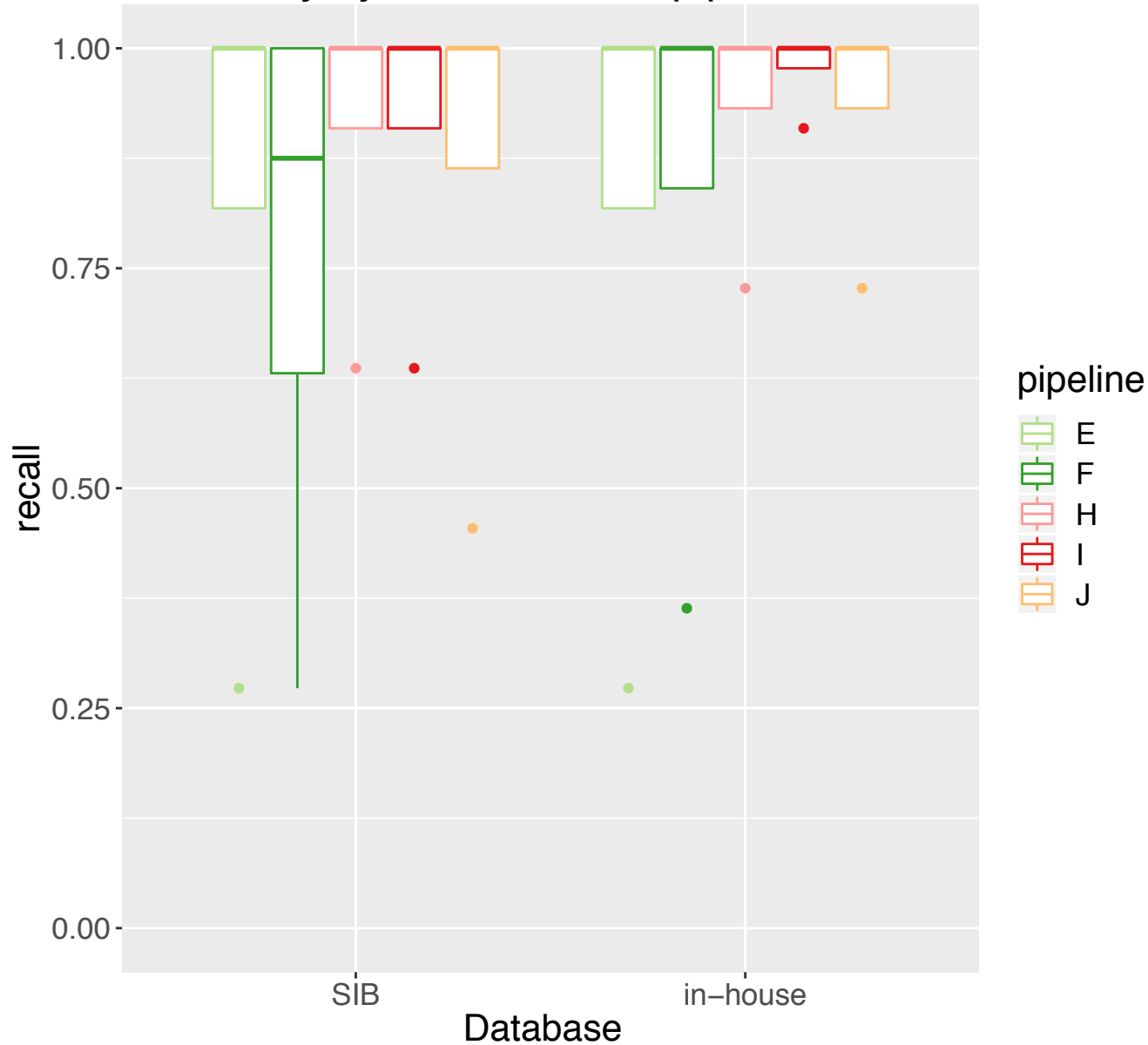

Supplement: Supplementary file 1 [file genes-10-00655-s001.zip › revised Suppl Material/Figure S21.pdf]

Precision by database and pipeline

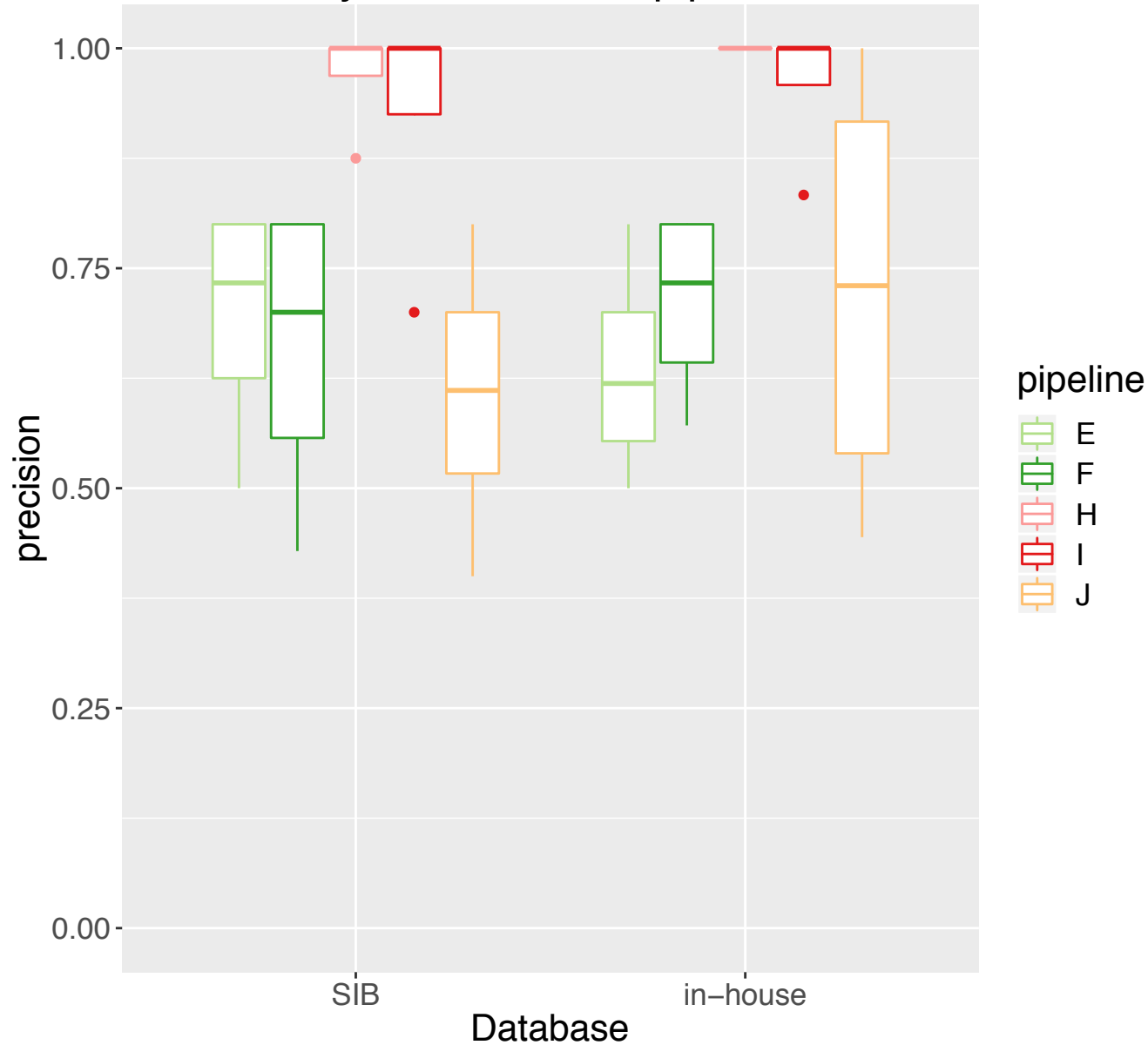

Supplement: Supplementary file 1 [file genes-10-00655-s001.zip › revised Suppl Material/Figure S20.pdf]

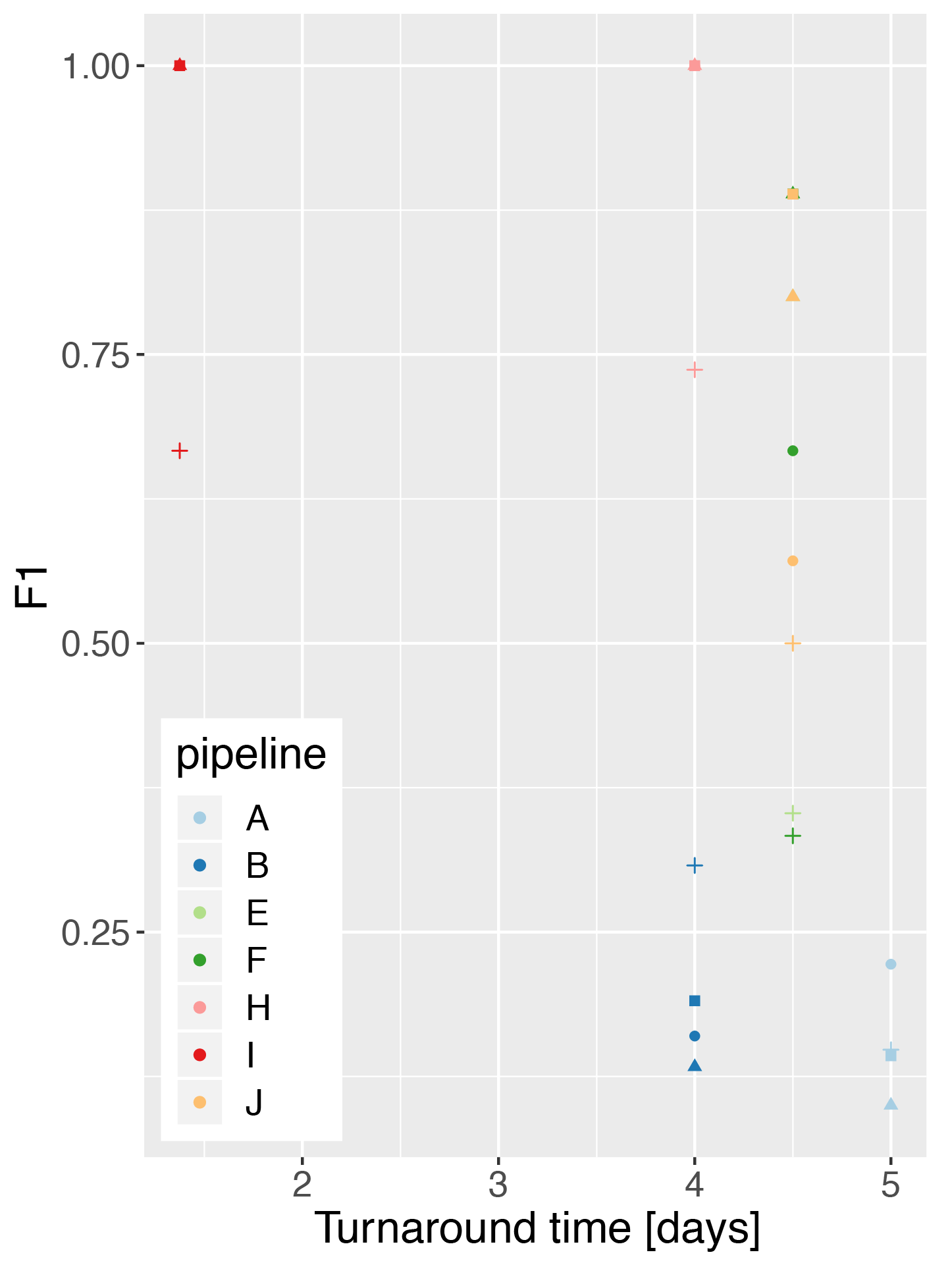

Supplement: Supplementary file 1 [file genes-10-00655-s001.zip › revised Suppl Material/Figure S2.png]

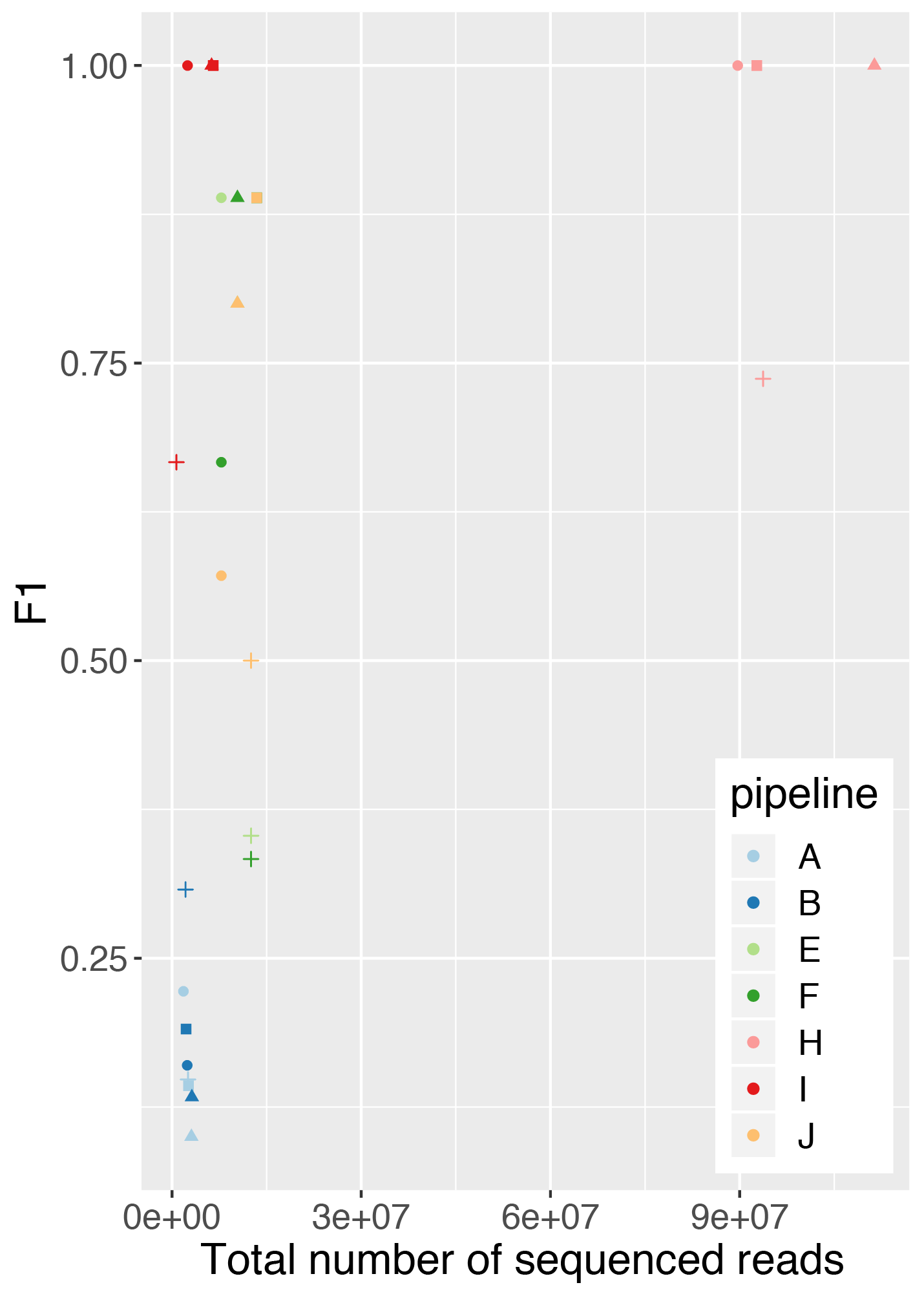

Supplement: Supplementary file 1 [file genes-10-00655-s001.zip › revised Suppl Material/Figure S1.png]
